# Specific volatiles of tea plants determine the host preference behavior of *Empoasca onukii*

**DOI:** 10.3389/fpls.2023.1239237

**Published:** 2023-08-31

**Authors:** Feng Chen, Peng Huang, Jun Wang, Wei Wu, Yong-Wen Lin, Jin-Feng Hu, Xin-Gang Liu

**Affiliations:** ^1^ Institute of Plant Protection, Fujian Academy of Agricultural Sciences/Fujian Key Laboratory for Monitoring and Integrated Management of Crop Pests/ Fujian Engineering Research Center for Green Pest Management, Fuzhou, China; ^2^ College of Food Engineering, Zhangzhou Institute of Technology, Zhangzhou Institute of Technology, Zhangzhou, China; ^3^ Institute of Plant Protection, Chinese Academy of Agricultural Sciences, Beijing, China

**Keywords:** *Camellia sinensis*, volatile organic compounds (VOCs), *Empoasca onukii*, behavioral preference, VOC synthesis-related genes

## Abstract

*Empoasca onukii* is a major pest that attacks tea plants. To seek effective and sustainable methods to control the pest, it is necessary to assess its host preference among different species of tea and understand the critical factors behind this behavior. In this study, the behavioral preference of *E. onukii* for volatile organic compounds (VOCs) of three potted tea species was evaluated. The VOCs released by the three tea species were analyzed using gas chromatography-mass spectrometry, and the major components were used to test the pest’s preference. Transcriptome analysis was used to infer the key genes that affect the biosyntheses of the VOCs. The results showed that the tendency of *E. onukii* toward the VOCs of the three tea species was the strongest in green tea, followed by white tea, and the weakest in red tea. This behavioral preference was significantly and positively correlated with the relative levels of hexanol, linalool, and geraniol in tea volatiles. Relative hexanol was significantly and positively correlated with the expression of genes *TEA009423 (LOX2.1)*, *TEA009596 (LOX1.5)*, *TEA008699 (HPL)*, *TEA018669 (CYPADH)*, and *TEA015686 (ADHIII)*. Relative linalool was significantly and positively correlated with the expression of genes *TEA001435 (CAD)* and *Camellia_sinensis_newGene_22126 (TPS)*. Relative geraniol was significantly and positively correlated with the expression of genes *TEA001435 (CAD)*, *TEA002658 (CYP76B6)*, *TEA025455 (CYP76T24)*, and *Camellia_sinensis_newGene_22126 (TPS)*. The above findings suggested that three volatiles (hexanol, linalool, and geraniol) determined the behavioral preference of *E. onukii* toward tea plants, and their biosynthesis was mainly affected by nine genes (*TEA009423*, *TEA009596*, *TEA008699*, *TEA018669*, *TEA015686*, *TEA001435*, *TEA002658*, *TEA025455*, and *Camellia_sinensis_newGene_22126*).

## 1 Introduction

Tea is one of the most popular nonalcoholic beverages worldwide, with an attractive aroma and pleasant taste. China is the birthplace of *Camellia sinensis* and is the largest producer and consumer of tea in the world. In 2022, the global tea cultivation area, production, and export volume were 4.37 million hectares, 6.397 million tons, and 1.827 million tons, respectively, whereas in China they were 2.65 million hectares, 3.35 million tons, and 0.375 million tons, respectively, which accounts for 60.6%, 52.4% and 20.5% of the global tea cultivation area, production and export volume, respectively (Hua, 2023; National Bureau of Statistics, 2023). Fujian is the largest tea-producing and exporting province in China, and among the main tea varieties grown are green tea (GT), red tea (RT), and white tea (WT). In 2022, the tea production and export volume of this province accounted for 15.54% and 25.52% of the national tea production, respectively, and the output value of the whole tea industry chain exceeded 150 billion yuan (Fujian Provincial Bureau of Statistics, 2023; Hua, 2023). In recent years, the expansion of tea planting has increased the occurrence of *Empoasca onukii*, which has become one of the main pests of tea. It can reduce the yield of tea by 20–30% in general and by at least 50% in severe cases, and may even cause extinctions; this seriously affects the yield and quality of tea and needs urgent prevention and control (Zhang et al., 2022). At present, *E*. *onukii is* controlled mainly by using chemical pesticide sprays and accounts for 40–60% of the total pesticide use in tea plantations (Zhang et al., 2020a). However, the frequent use of chemical pesticides leads to residues, resistance, and re-emergence. The “push-pull” pest management strategy is a novel and efficient integrated pest management tool that can use color (visual), chemical pheromones (olfactory), and sound (acoustic) as “push” or “pull” components (Cook et al., 2007). Based on the selection behavior of pests for plant volatiles, repellents that protect target plants or lures to attract target pests have been developed in the integrated pest management of *Leptinotarsa decemlineata* (Martel et al., 2005), *E. vitis* (Mu et al., 2012; Zhang et al., 2014), *E. flavescens* (Niu et al., 2022), and *Frankliniella occidentalis* (Kim et al., 2023). However, the application of the “push-pull” strategy in the control of *E. onukii* has not been reported so far. This is mainly because the selection behavior of *E. onukii* on host plants such as tea leaves is unclear, which limits the development and application of lures or repellents for this insect. Therefore, there is an urgent need to conduct relevant research to support the subsequent green and sustainable control of this insect.

Many factors are known to determine the host selection of phytophagous insects, including secondary metabolites produced by plants (Kong and Lou, 2010). Volatile organic compounds (VOCs) released by plants are small compounds with low boiling points that are produced through secondary metabolic pathways (Dudareva et al., 2013; Goh et al., 2016). The volatiles released by different plants vary greatly and play a crucial role in plant–insect interactions (Mody et al., 2015; Sun et al., 2019; Sharma et al., 2021). Evidence indicates that VOCs are chemical cues for phytophagous insects to search for host plants, as well as self-defense weapons for plants to defend themselves against phytophagous insects (Bruce et al., 2005; D’Alessandro and Turlings, 2005 and Dudareva and Negre, 2005; D’Alessandro and Turlings, 2006; Tholl, 2006; Hare, 2011; Poelman et al., 2012; Sun et al., 2012; Anderson and Anton, 2014). Therefore, the search for plant volatiles with lure or repellent functions for phytophagous insects and the identification of genes related to the synthesis of these specific volatiles are of great significance in the development of specific lures or repellents for pests and the cultivation of insect-resistant varieties using genetic engineering technology for the development of green pest control.

How *E. onukii* selects volatiles from different tea varieties, the specific components of the volatiles involved in these behavioral preferences or the genes involved in the synthesis of these components is unclear. Resolving these problems can provide a basis for the development of specific lures or repellents for *E. onukii*, as well as the breeding of tea varieties resistant to *E. onukii* through genetic engineering technology. This could not only effectively control the damage by the insect and improve the quality and yield of tea but also promote the application of green pest control measures, reduce the use of chemical pesticides, and promote the green development of the tea industry, with significant economic, social and ecological benefits. In this study, we selected potted green, red, and white tea grown in Fujian as the test plants to observe the behavioral preference of *E. onukii* to their volatiles. The components and contents of the three tea volatiles were measured, and the correlation between the behavioral preference of the insect and the tea volatiles was analyzed to detect the specific volatile components that determine the selection behavior of the insect. Then, the behavioral preference of *E. onukii* to the standards of these specific volatile components was observed to verify the specificity of each volatile component. Finally, we analyzed the transcriptome changes of the three tea species to find the genes related to the synthesis of specific volatiles. This study could provide key components for the subsequent development of specific attractants or repellents of *E. onukii* and candidate target genes for the breeding of resistant tea varieties using genetic engineering technology to promote the development of green pest control.

## 2 Materials and methods

### 2.1 Materials

#### 2.1.1 Test plants

GT ‘Jiulongcuiya’, RT ‘Jinmudan’, and WT ‘Jiulongdabai’ were selected from the tea planting base in Nanping City, Fujian Province (27.48° N, 118.82° E) with potted tea seedlings plants 20–22 cm in height, then the seedlings were transferred to 12 cm (diameter) × 10 cm (height) × 8 cm (bottom diameter) pots with peat soil and restored for 10 d (**Figure S1**).

#### 2.1.2 Test insects

*E. onukii* was collected from the same tea planting site as above and grown at room temperature 24–27°C, relative humidity 55%–65%, and photoperiod 16L:8D. The adults in the next generation fledged for 2 d of the second generation were starved for 12 h and kept in cages.

### 2.2 Methods

#### 2.2.1 Determination of the behavioral preference of E. onukii to tea VOCs

The behavioral preference of *E. onukii* to volatiles from GT, RT, and WT was analyzed in a two-way selection test using a Y-type olfactometer at a room temperature of 24–27°C and in darkness and confinement, following the methods of Lin et al. (2016) and Niu et al. (2022). Six groups were set in the experiment: (1) GT vs. fresh air (FA), (2) RT vs. FA, (3) WT vs. FA, (4) GT vs. RT, (5) GT vs. WT, and (6) RT vs. WT; each treatment was replicated three times.

**Figure 1:**
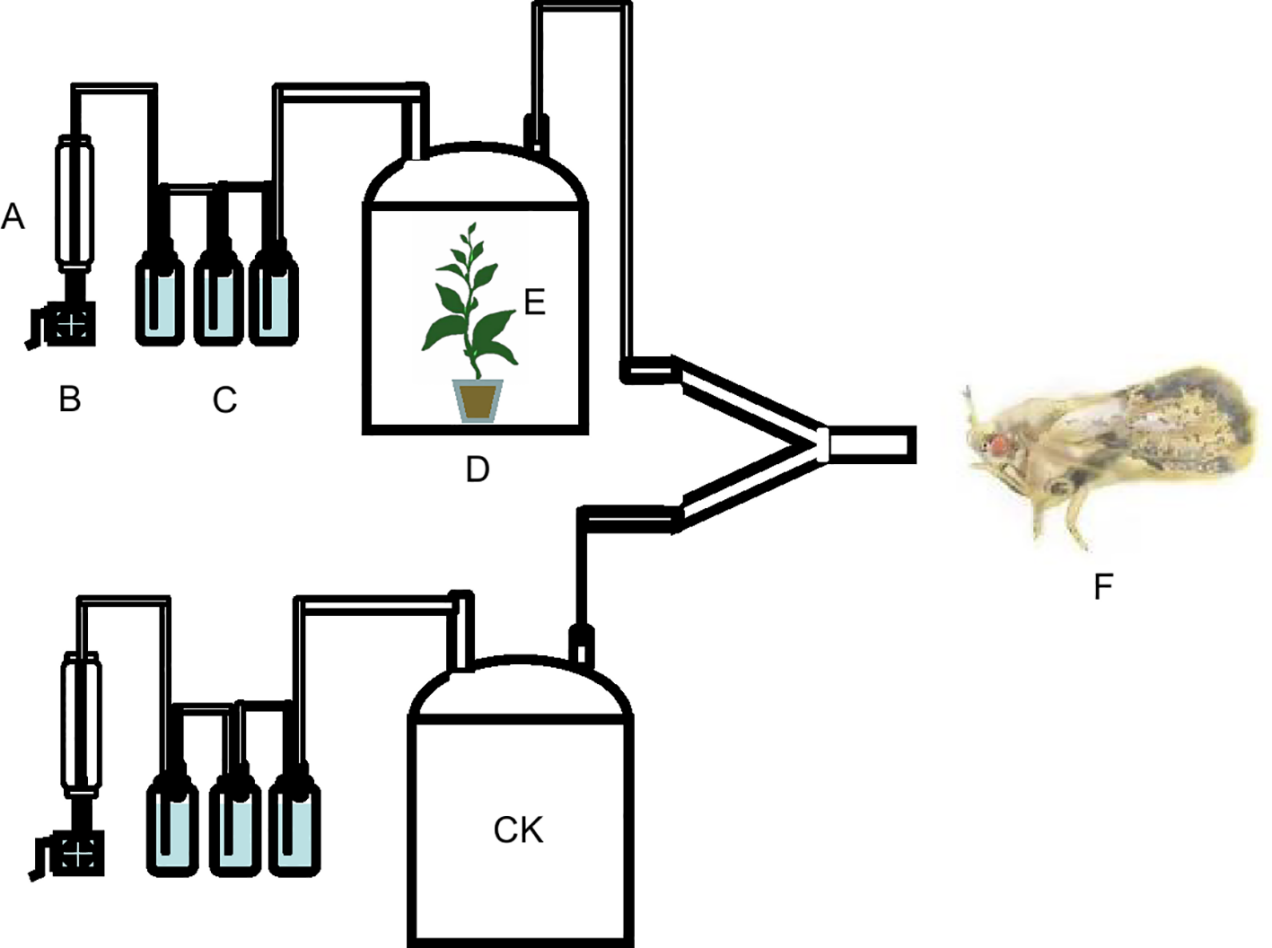
Schematic diagram of the olfactory experiment of *Empoasca onukii* to natural volatile organic compounds in three potted tea plants. **(A)** Flow meter, **(B)** pump, **(C)** air-purifying cups, **(D)** glass jar, **(E)** tea plants, and **(F)** *Empoasca onukii* adult, (CK) Fresh air/Tea plants.

The three different potted tea seedlings were sealed in seedling pots and peat soil under polyethylene cling film and placed in a 35 cm (height) × 25 cm (diameter) glass air collector for 24 h. Then fresh air was blown by an air pump at a flow rate of 200 ± 10 mL/min and filtered by a molecular sieve, color-changing silica gel, and activated carbon, through the air inlet at the top of the air collector and blown out through the air outlet along the top of the air collector, then into the Y-type olfactometer. A small amount of cotton was inserted at the interface between the two arms of the olfactometer and the silicone tube to prevent *E. onukii* from crawling into the collection tank along the hose during the test (**Figure 1**). An adult *E. onukii* was placed in the entrance of the main arm of the Y-type olfactometer, and then the entrance was blocked with cotton to prevent the insect from escaping. The selection was considered valid if *E. onukii* crawled into more than 1/3 of both arms of the Y-type olfactometer within 5 min and continued for more than 30 s. If *E. onukii* did not select after 5 min, the selection was considered abandoned. Each *E. onukii* was used only once in the experiment, and the Y-type olfactometer was changed every 5 insects. The number of effective selections of *E. onukii* in each treatment was counted at the end of each group, and the Y-type olfactometer, silicone tubes, and glass collection cylinders were washed with ethanol and hot water at 60–70°C. Student’s *t*-test in GraphPad Prism 7 (GraphPad Software Inc., San Diego, CA, USA) was then used to compare the differences in olfactory responses of *E. onukii* in each treatment.

#### 2.2.2 Determination of VOCs in tea leaves and its correlation with the behavioral preference of E. onukii

The volatiles of the three tea species were extracted by solid-phase microextraction (SPME) and detected by gas chromatography-mass spectrometry (GC-MS) (7890B-5977A; Agilent, Palo Alto, CA, USA), referring to Mei et al. (2017); Vallarino et al. (2018) and Niu et al. (2022). Three seedlings of the three different tea plants were taken at room temperature of 24–27 °C and the leaves of each plant were removed and immediately fixed in liquid nitrogen for 15 min to prevent the volatiles from changing due to trauma. Then, 5 g of each tea plant was randomly taken and placed into a 100 mL headspace vial. A 57328-U solid-phase microextraction needle (Sigma-Aldrich, St. Louis, MO, USA) was inserted into the headspace vial. The extraction was performed for 60 min and volatiles were detected on the machine.

Chromatographic column: An HP-5MS capillary column (30 mm × 0.25 mm, i.d., 1.0 µm film thickness, Agilent); inlet temperature 250 °C, interface temperature 280 °C; programmed temperature rise: initial temperature 50 °C for 5 min, then 3.0 °C/min to 190 °C for 5 min, then 8.0 °C/min to 200 °C for 5 min, then 15.0 °C/min to 280 °C for 15 min. Mass spectrometry conditions were an EI ionization electron energy of 70 eV. Mass spectra were acquired by scanning from 25–500 m/z. Based on the retention time of each volatile substance in the chromatogram during the detection of tea samples, the NIST library of GC-MS was searched, and the search results were checked with references to the standard spectra. The components of the volatiles from the three tea varieties were identified and analyzed, and the relative content of each component was calculated by the normalization method of peak area. The Tukey’s test in GraphPad Prism 7 software was used to compare the differences in the relative contents of the volatile components of the three tea leaves. The effective selection number of *E. onukii* on the three tea leaves and the relative contents of each volatile component among the three tea leaves were used as variables. The correlation between two variables was analyzed by Pearson correlation and a significance test *p* < 0.05 was used as the criterion for screening the specific components that determine the host selection behavior of *E. onukii*.

#### 2.2.3 Determination of the behavioral preference of E. onukii to volatile component standards

Referring to the methods of Lin et al. (2016) and Niu et al. (2022), hexanol, linalool, and geraniol, were selected from tea volatiles that showed a significant correlation with the behavioral preference of *E*. *onukii.* The three components (97–99% purity) were purchased from Shanghai Macklin Biochemical Co., Ltd. Each component was dissolved in triethyl citrate (98% purity), a slow-release solvent, and diluted to different concentrations before the test.

In the single component treatment, the concentration of each component standard was based on its relative content in the three tea volatiles, including 2, 4, and 6 µg/100 µL for hexanol, 4, 8, and 12 µg/100 µL for linalool, and 3, 6 and 9 µg/100 µL for geraniol. Air was used as blank control for each treatment. In the mixed component treatment, the concentration ratio of each component standard was set to simulate the relative content of the corresponding volatiles in the three tea species. The GT blend contained 3.97 µg/100 µL of hexanol, 8.56 µg/100 µL of linalool, and 5.65 µg/100 µL of geraniol, the RT blend contained 0.91 µg/100 µL of hexanol, 2.18 µg/100 µL of linalool and 1.37 µg/100 µL of geraniol, and the WT blend contained 2.10 µg/100 µL of hexanol, 4.64 µg/100 µL of linalool and 3.51 µg/100 µL of geraniol. Three sets of comparison treatments were set up for the three mixed components: (1) blend GT vs. blend RT, (2) blend GT vs. blend WT, and (3) blend RT vs. blend WT. After that, 1 mL of the suspension solution of each single ingredient or blend of ingredients for each treatment was pipetted onto a small piece of cotton; then the small piece of cotton was placed in an open glass Petri dish, and then the Petri dish was placed in an air collection jar, after which the effective selection number of *E. onukii* in each treatment was observed and counted according to the previous method. Thirty *E. onukii* were observed in each treatment, and this was repeated three times. The t-test in GraphPad Prism 7 software was used to compare the differences in the behavioral preference of *E. onukii* in each treatment of the three compound standards to verify the specificity of the three volatile components.

#### 2.2.4 Expression analysis of genes related to the synthesis of VOCs in tea leaves

Transcriptome analysis: Leaves from three seedlings of the three different tea plants were cut quickly and immediately fixed in liquid nitrogen for 15 min to prevent transcriptional changes caused by trauma. Then, 5 g of leaves from each potted tea plant were randomly taken into 50 mL lyophilization tubes, immediately buried in dry ice, and sent for total RNA extraction, library construction, and sequencing. Total RNA was analyzed by Nanodrop 1000 ultra-micro spectrophotometer (Thermo Fisher, Waltham. MA, USA), Qubit 2.0 fluorescence quantification instrument, and Agilent 2100 Bioanalyzer. After library construction, high-throughput sequencing was performed using an Illumina HiSeq 2000 (Illumina, SanDiego, CA, USA). After the sequencing and datas quality control were completed (**Tables S1**-**S3**), the *Camellia sinensis* genome (http://tpdb.shengxin.ren/) was used as the reference, and the HISAT2 system and StringTie software were applied to compare and assemble the valid data volume (clean reads) obtained from the sequencing, and the Unigene sequences obtained were compared with BLASTx on NCBI NR, Swiss-Prot, GO, COG, KOG, EggNOG, Pfam, and KEGG databases. Those with similarity > 30% and e < 1e^-5^ were used for Unigene annotation and adjusted with the Benjamini-Hochberg method. Using the Benjamini-Hochberg method, some genes related to the synthesis of three specific volatiles were initially selected based on the false discovery rate (FDR) < 0.01 and fold change |(FC)| > 2, and the functional annotation of the genes. Then the expression of these related genes was normalized (Benjamini and Hochberg, 1995; Benjamini and Yekutieli, 2001), and the correlation between the three specific volatile components and the expression of the related genes was analyzed by Pearson’s correlation in GraphPad Prism 7 software, followed by a significance test of *p*< 0.05 was used as the criterion to screen the key genes.

Real-time quantitative fluorescence PCR (RT-qPCR) validation: Referring to the method of Fang et al. (2022), we designed the specific primers (**Table S4**) for genes related to the synthesis of three specific volatile components using Primer Premier 5.0 software, using β-actin as the internal reference gene, and the cDNA was synthesized using the TRUEscript 1st Strand cDNA Synthesis Kit Reverse Transcription Kit (Beijing Aidlab Biotechnologies Co. Ltd., Beijing, China). The amplified products were detected on a qTOWER2.2 real-time fluorescence PCR system (Analytikjena, Jena, Germany) using 2 × SYBR Green Premix (Chengdu Danfeng Technology Co. Ltd., Chengdu, China)), and repeated three times for each sample. The relative expression of the genes related to the synthesis of the three specific volatile components was calculated using the 2^−ΔΔCT^ method (Livak and Schmittgen, 2001).

## 3 Results

### 3.1 Behavioral preference of E. onukii to tea VOCs

**Figure 2:**
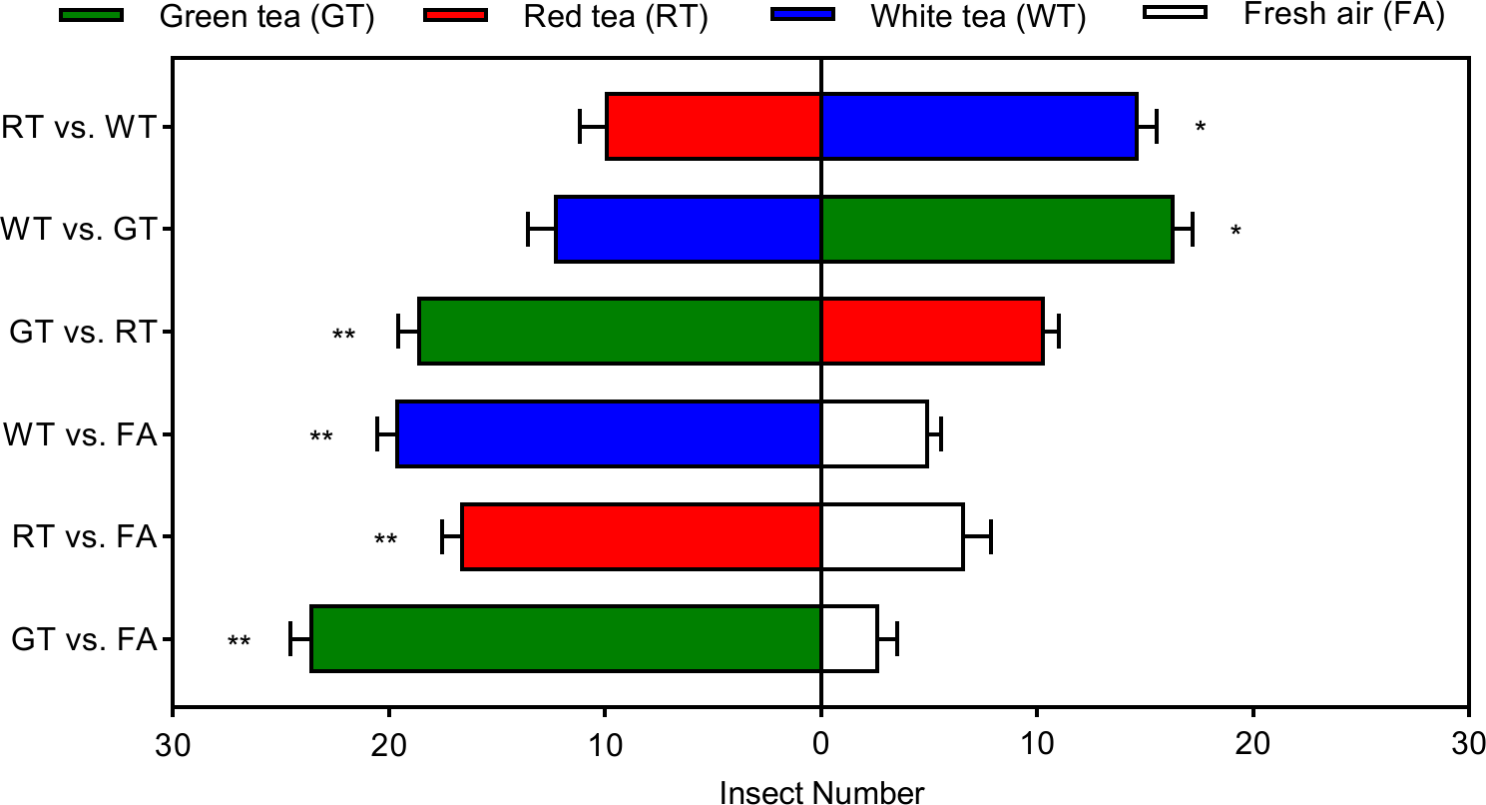
Behavioral preference of *Empoasca onukii* to natural volatile organic compounds in three potted tea plants. Bars indicate mean±SE. The * and ** indicate significant difference (*P<0*.05) and highly significant difference (*P*<0.01), respectively.

The effective selection numbers of *E. onukii* for the three tea tree volatiles were significantly different. The highest effective selection numbers were for GT, GT vs. FA, GT vs. RT, and GT vs. WT were 23. 67, 18.67, and 16.33, respectively. The effective selection numbers for GT were significantly more than for air (*t*=16.8375, *p*=0.0001, *df*=4), RT (*t*=7.5378, *p*=0.0017, *df*=4), and WT (*t*=3.2071, *p*=0.0327, *df*=4). The effective number of selection on WT was the next highest, WT vs. FA, RT vs. WT, and GT vs. WT at 19.67, 14.67, and 12.33, respectively, and significantly more than for air (*t*=13.9140, *p*=0.0002, *df*=4) and RT (*t*=3.2118, *p*=0.0325, *df*=4). The least effective number of selections was on RT, which was only 16.67, 10.33, and 10.00 in RT vs. FA, GR vs. RT, and RT vs. WT, respectively, but significantly higher than air (*t*=6.7082, *p*=0.0026, *df*=4) (**Figure 2**).

### 3.2 Correlation between VOCs of tea leaves and behavioral preference of E. onukii

**Figure 3:**
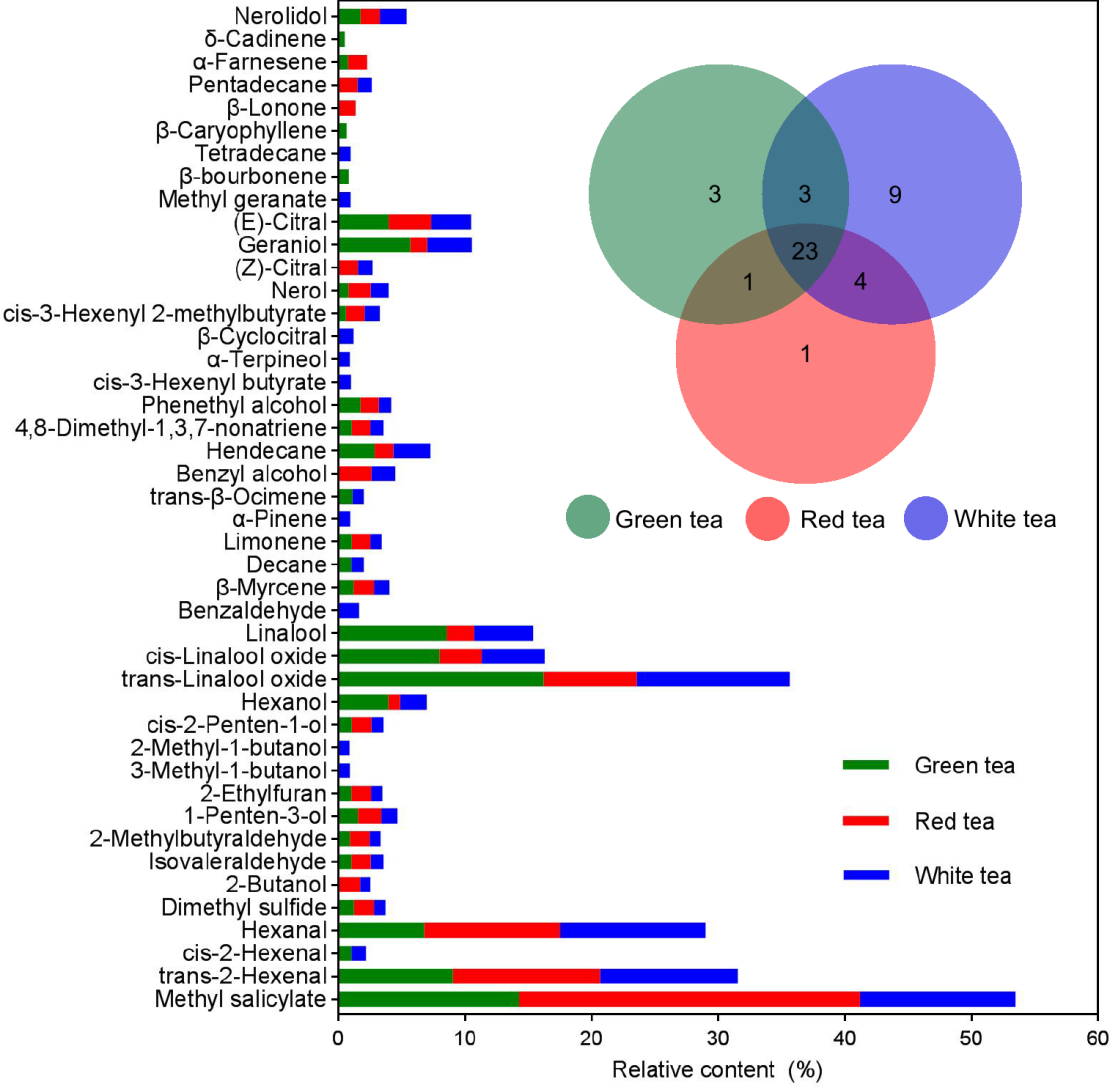
Relative content and Venn set of volatile organic compounds (VOCs) in three potted tea plants. Different color columns and circles indicate relative content and the number of the digits in different color circles indicates unique (nonoverlapping color parts) and shared (overlapping color parts) VOC number among three potted tea plants. The digits in different color circles indicate unique (nonoverlapping color parts) and shared (overlapping color parts) VOC number among three potted tea plants.

A total of 44 volatile components were identified from the three tea species, and the number of volatiles was not significantly different among the three species, including 30 for GT, 29 for RT, and 39 for WT. The compositions were also similar, with 24 volatiles identical between GT and RT, 26 volatiles identical between GT and WT, 27 volatiles identical between RT and WT, and 23 volatile components identical among the three species (**Figure 3**). However, the relative contents of each component in the three tea volatiles were different, among which the relative contents of hexanol, linalool, and geraniol were not high, ranging from 0.91% to 3.97%, 2.18% to 8.56%, and 1.37% to 5.65%, respectively, but the differences were significant among the varieties (F=12.0810–137.1140, *p*=0.0001–0.0079, *df*=8), and all showed significant positive correlations (*r*≥0.9976, *p*<0.05) with the behavioral preference of *E*. *onukii* (**Figure 3**, **S2**; **Tables 1**, **S5**).

**Table 1:** Correlations between relative content of volatile organic compounds of tea plants and behavioral preference of *Empoasca onukii* (mean ± SE, *p* < 0.1).

VOCs component	Relative content (%)			*r*	*p*
	Green tea	Red tea	White tea		
2-Butanol 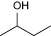	0.00 ± 0.00^c^	1.73 ± 0.04^a^	0.81 ± 0.01^b^	-0.9992	0.0625
Hexanol 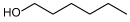	3.97±0.19^a^	0.91±0.12^c^	2.10±0.06^b^	0.9978	0.0421^*^
trans-Linalool oxide 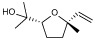	16.22±0.20^a^	7.33±0.30^c^	12.09±0.20^b^	0.9948	0.0651
cis-Linalool oxide 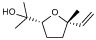	7.98±0.16^a^	3.36±0.02^c^	4.97±0.02^b^	0.9984	0.0712
Linalool 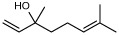	8.56±1.24^a^	2.18±0.25^c^	4.64±0.34^b^	0.9976	0.0445^*^
cis-3-Hexenyl 2-methylbutyrate 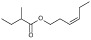	0.58±0.02^c^	1.50±0.05^a^	1.17±0.04^b^	-0.9996	0.0639
Nerol 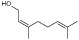	0.79±0.03^c^	1.77±0.02^a^	1.43±0.07^b^	-0.9936	0.0723
Geraniol 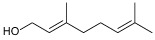	5.65±0.72^a^	1.37±0.29^c^	3.51±0.73^b^	0.9981	0.0391^*^

Different lowercase letters in the same row and *indicate significant differences (p < 0.05). The r indicates Pearson’s correlation coefficient.

### 3.3 Behavioral preference of E. onukii to volatile component standards

**Figure 4:**
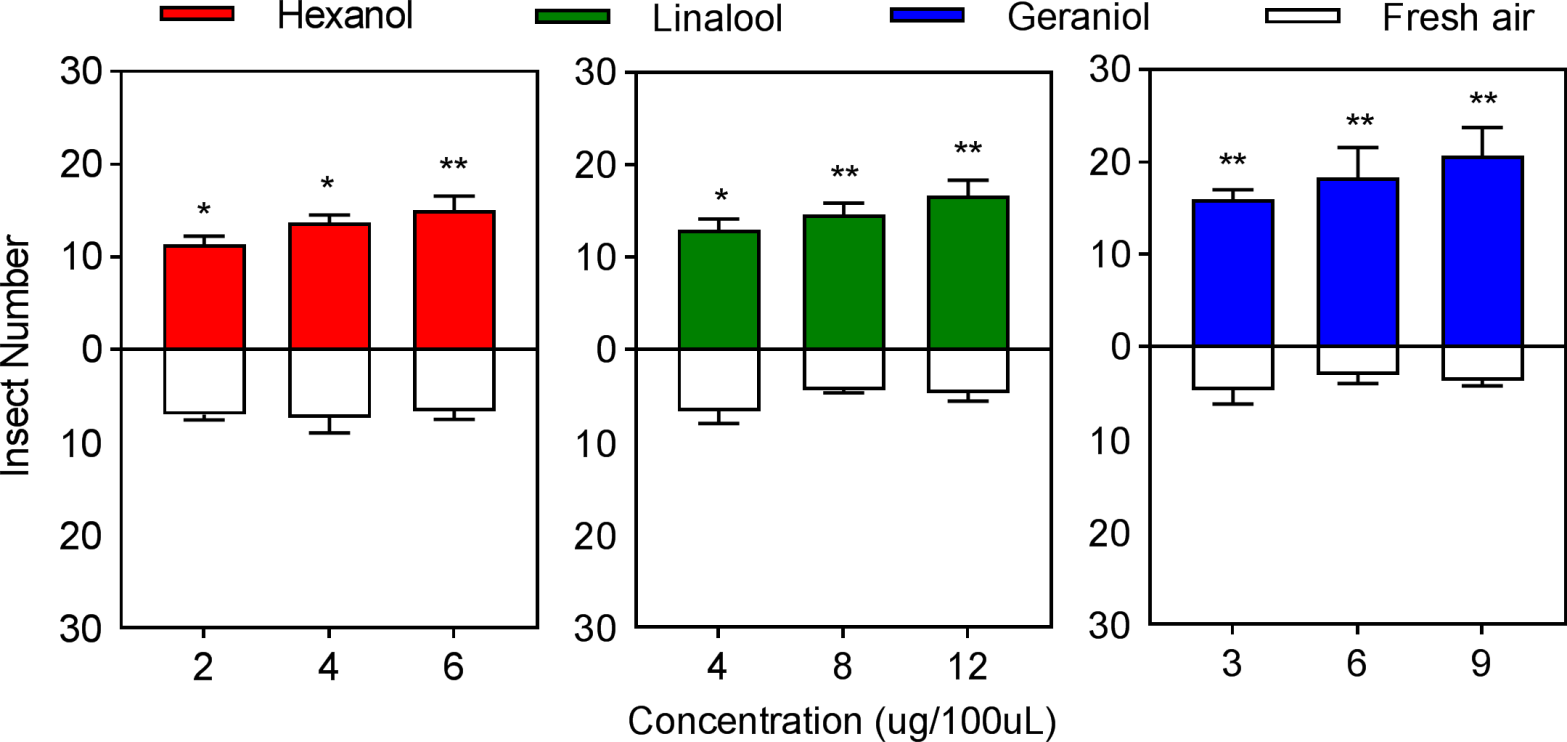
Behavioral preference of *Empoasca onukii* to three volatile organic compounds. Bars indicate mean±SE. The * and ** indicate significantly different parameters (*P<0*.05) and bars indicate the mean±SE; the * and ** indicate significantly different (*P*<0.05) and highly significantly different parameters (*P*<0.01), respectively.

The behavioral preference of *E. onukii* to the three volatile constituent standards was strongly influenced by the concentrations. The effective selection number of *E. onukii* increased with increasing volatile concentration in the experimental design concentration range of a single constituent. In hexanol, the effective selection number was significantly higher for 2 µg/100 µL (*t*=4.1110, *p*=0.0283, *df*=4) and 4 µg/100 µL (*t*=3.3588, *p*=0.0147, *df*=4) than for air, and the effective selection number was significantly higher for 6 µg/100 µL (*t*=4.7246, *p*=0.0091, *df*=4) than air. In linalool, the effective selection number of *E. onukii* was significantly higher for 4 µg/100 µL (*t*=3.5907, *p*=0.0229, *df*=4) than air, and the effective selection number of *E. onukii* for 8 µg/100 µL (*t*=8.2851, *p*=0.0012, *df*=4) and 12 µg/100 µL (*t*=6.3640, *p*= 0.0031, *df*=4) was significantly higher than air. In geraniol, 3 µg/100 µL (*t*=10.7517, *p*=0.0004, *df*=4), 6 µg/100 µL (*t*=7.8889, *p*=0.0014, *df*=4), and 9 µg/100 µL (*t*=9.4705, *p*=0.0007, *df*=4) were significantly higher than the control (**Figure 4**).

**Figure 5:**
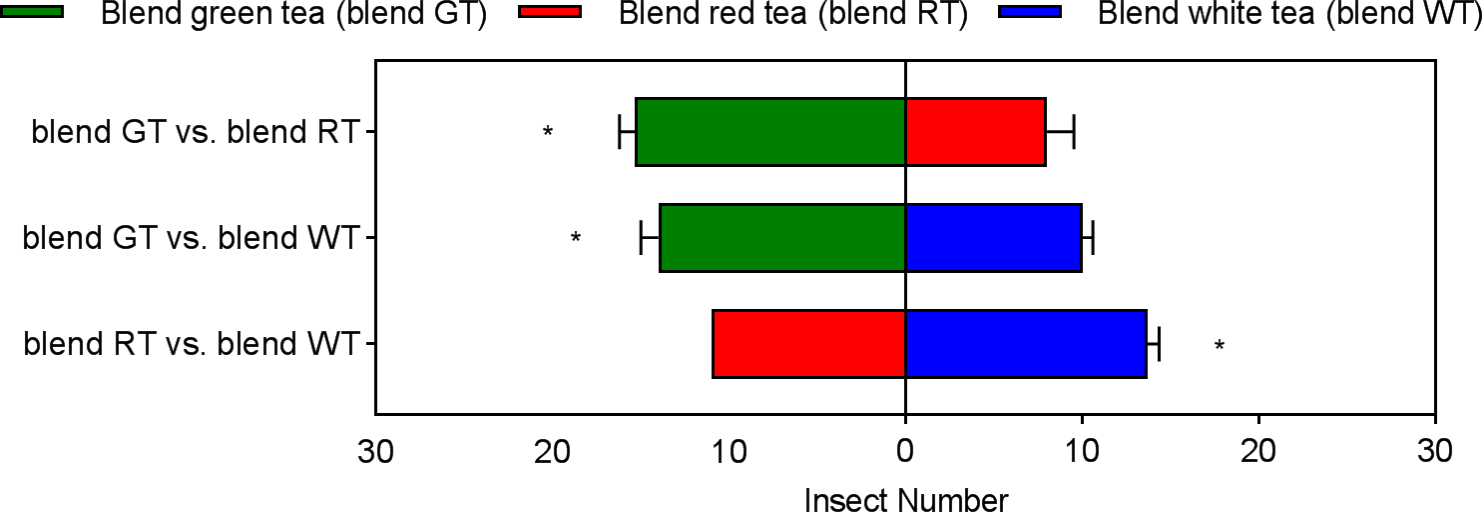
Behavioral preference of *Empoasca onukii* to three volatile blends. Bars indicate the mean±SE. The * indicates a significant difference (*P*<0.05).

There was also a significant difference in the number of effective selections of *E. onukii* among the experimental design treatments for the volatile blend components. *E. onukii* had the highest number of effective selections in the GT blend, which was significantly higher than in the RT blend (*t*= 4.1576, *p*=0.0142, *df*=4) and WT blend (*t*= 3.4641, *p*=0.0257. *df*=4). *E. onukii* had the next highest number of effective selections in the WT blend, significantly more than in the RT blend (*t*=3.0237, *p*=0.0390, *df*=4). The lowest number of effective *E. onukii* selections occurred in the RT blend (**Figure 5**). It can be seen that the behavioral preference of *E. onukii* for synthetic and natural volatiles was similar.

### 3.4 Expression of genes related to the synthesis of VOCs in tea leaves

**Figure 6:**
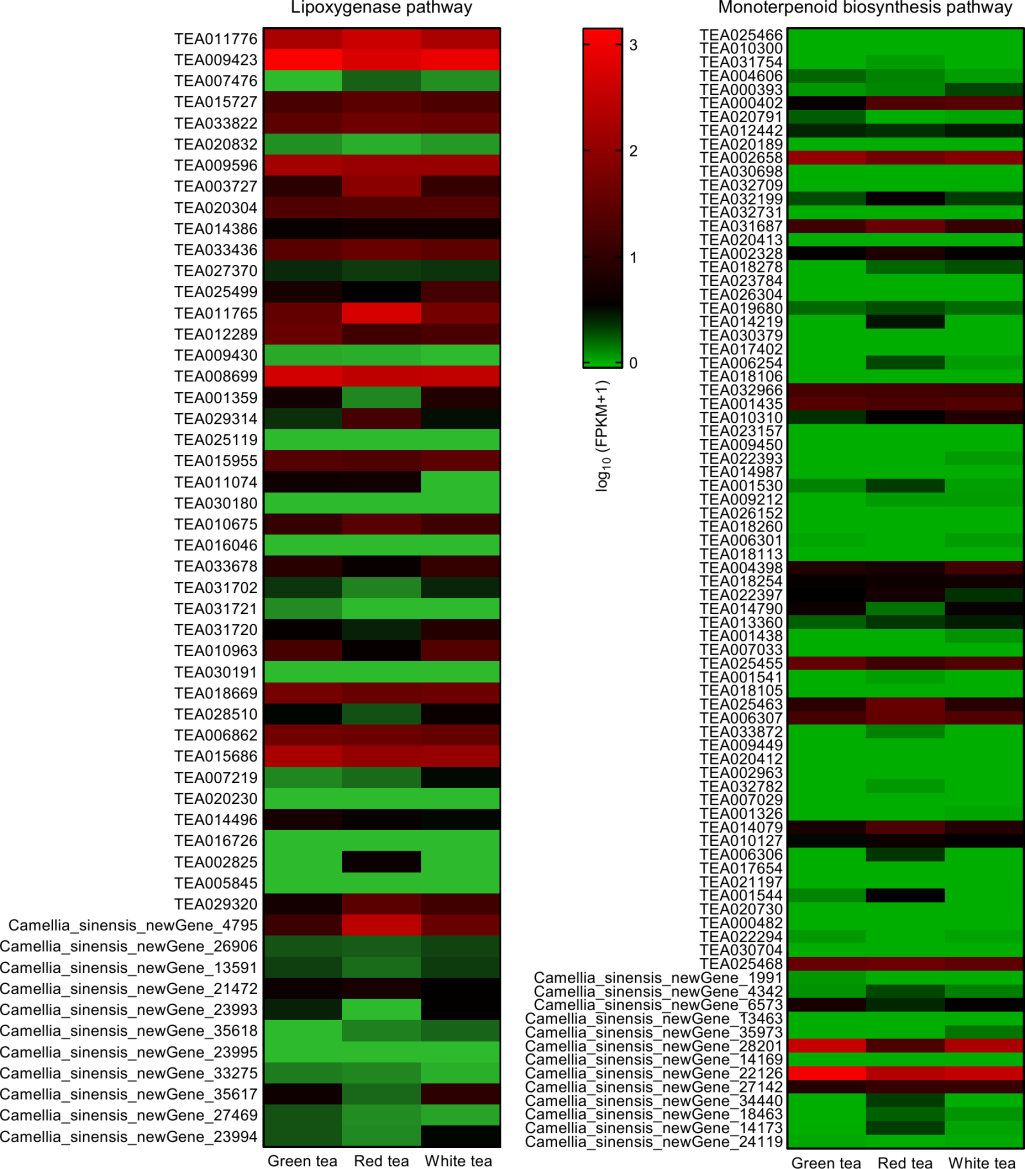
Expression heat map of lipoxygenase genes and monoterpene synthase genes of leaves in three potted tea plants. FPKM, fragments per kilobase of exon per million mapped fragments.

Transcriptome analysis of the leaves of GT, RT, and WT revealed that there were 53 differentially expressed genes directly involved in hexanol synthesis (lipoxygenase pathway) and 82 differentially expressed genes directly involved in linalool and geraniol synthesis (monoterpenoid biosynthesis pathway) in the three tea species (**Figure 6**).

**Figure 7:**
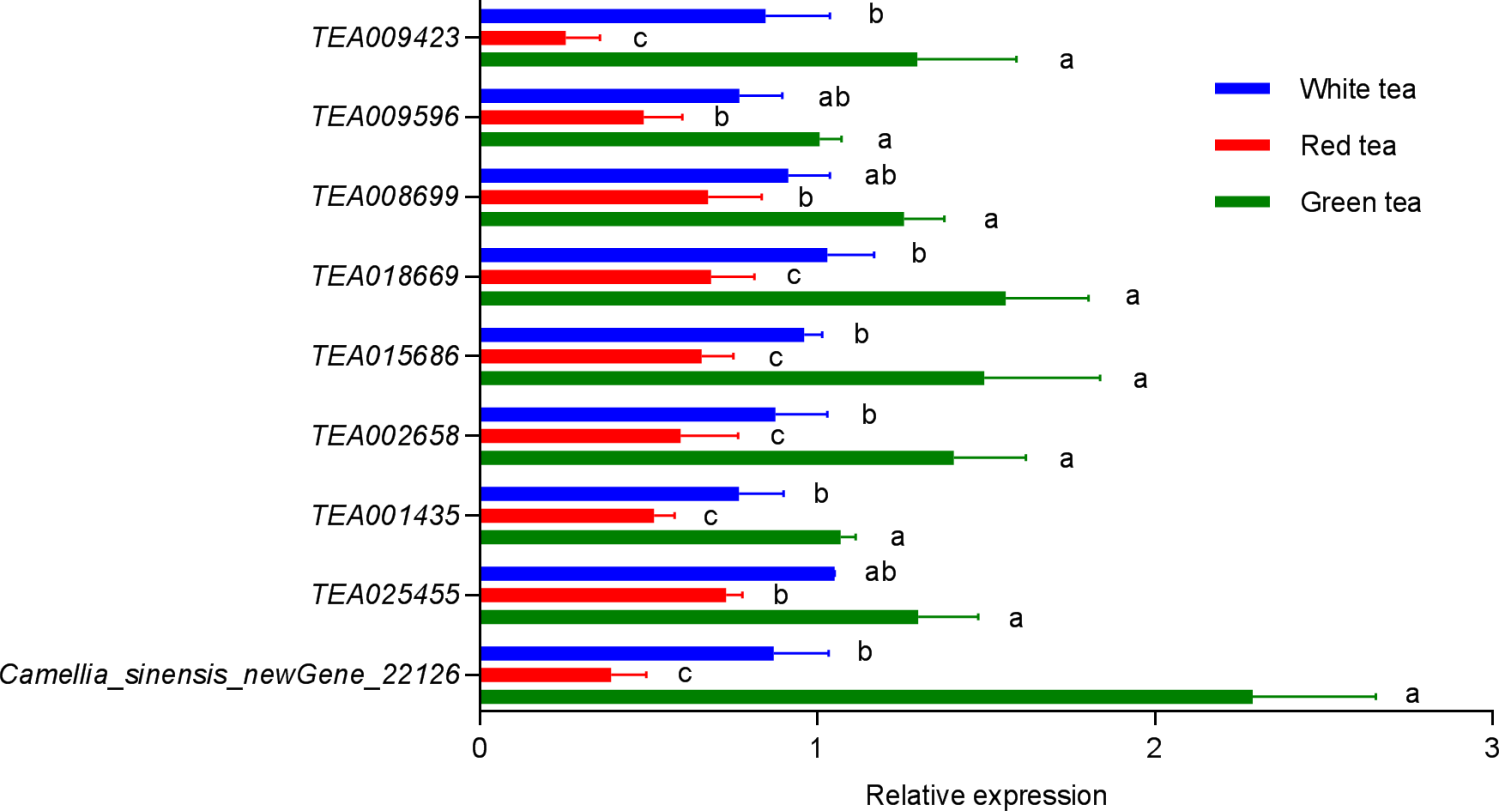
Relative expression of nine genes related to volatile synthesis in three potted tea plants. Bars indicate the mean±SE. Different lowercase letters indicated a significant difference (*P*<0.05).

Combined with Pearson’s correlation analysis, the relative hexanol content in the three teas was found to be correlated with the genes in the lipoxygenase pathway *TEA009423 (LOX2.1)* (*r*=0.9981, *p*=0.0390, *df*=4), *TEA009596 (LOX1.5)* (*r*=0.9976, *p*=0.0443, *df*=4), TEA008699 (*HPL*) (*r*=0.9978, *p*=0.0423, *df*=4), *TEA018669 (CYPADH)* (*r*=0.9982, *p*=0.0379, *df*=4), and *TEA015686 (ADHIII)* (*r*=0.9992, *p*=0.0250. *df*=4) expression were consistent with each other and showed a significant positive correlation. The relative content of linalool was significantly correlated with the expression of the genes *TEA001435 (CAD)* (*r*=0.9978, *p*=0.0426, *df*=4) and *Camellia_sinensis_newGene_22126 (TPS)* (*r*=0.9978, *p*=0.0419, *df*=4) in the monoterpenoid biosynthesis pathway showed the same trend and a significant positive correlation. The relative content of geraniol was significantly correlated with the expression of genes *TEA001435 (CAD)* (*r*=0.9979, *p*=0.0410, *df*=4), *TEA002658 (CYP76B6)* (*r*=0.9999, *p*=0.0090, *df*=4), *TEA025455 (CYP76T24)* (*r*=0.9997, *p*= 0.0143, *df*=4), and *Camellia_sinensis_newGene_22126 (TPS)* (*r*=0.9979, *p*=0.0417, *df*=4) showed consistent and significant positive correlation in expression (**Tables 2**, **S6**). A q-PCR analysis also confirmed that the trends of relative expression of these nine volatile compound synthesis-related genes in the three tea species were consistent with the results of digital gene expression profiling (**Figure 7**).

**Table 2:** Correlations between relative content of three volatiles and expression of nine genes in three potted tea plants (*P*<0.1).

VOCs Name	Gene ID (Annotation)	*r*	*p*
Hexanol	*TEA009423 (LOX2.1)*	0.9981	0.0390^*^
	*TEA009596 (LOX1.5)*	0.9976	0.0443^*^
	*TEA008699 (HPL)*	0.9978	0.0423^*^
	*TEA018669 (CYPADH)*	0.9982	0.0379^*^
	*TEA015686 (ADHIII)*	0.9992	0.0250^*^
Linalool	*TEA001435 (CAD)*	0.9978	0.0426^*^
	*TEA002658 (CYP76B6)*	0.9894	0.0926
	*TEA025455 (CYP76T24)*	0.9882	0.0980
	*Camellia_sinensis_newGene_22126 (TPS)*	0.9978	0.0419^*^
Geraniol	*TEA001435 (CAD)*	0.9979	0.0410^*^
	*TEA002658 (CYP76B6)*	0.9999	0.0090^**^
	*TEA025455 (CYP76T24)*	0.9997	0.0143^*^
	*Camellia_sinensis_newGene_22126 (TPS)*	0.9979	0.0417^*^

The * and ** indicate significant difference (P<0.05) and highly significant difference (P<0.01), respectively. The r indicates Pearson’s correlation coefficient.

## 4 Discussion

Insects and plants have evolved together over a long period of time to form a variety of interactions, in which phytophagous insects express their predation in a series of behavioral responses, including insect orientation and recognition of host plants (Qin and Wang, 2001). It has been shown that volatiles released from plants can induce directional behavior of insects and are important stimulus signals for phytophagous insects to search for host plants. For example, Tingle et al. (1990) studied the selection of *Heliothis virescens* and *H. subflexa* female moths for insect-susceptible and insect-resistant host plants and found that *H. virescens* showed a tendency to volatiles from insect-susceptible *Nicotiana tabacum*, *Desmodium tortuosum*, Gossypium and Physais plants, while *H. subflexa* showed a significant tendency to volatiles from insect-susceptible Physais host plants, but both nocturnal moths showed no tendency to volatiles from insect-resistant tobacco species. In the present study, we found that the tendency of *E. onukii* to the volatiles of the three tea species was strongest in GT, followed by WT, and weakest in RT, which may be related to the difference in the resistance of tea species to *E. onukii*.

Plant volatiles are usually a mixture of secondary metabolites, consisting of fatty acid derivatives, terpenoids and their derivatives, sulfides, aromatic compounds, etc. (Bernays and Chapman, 1994; Belostotsky, 2009). The volatiles contained in different plants can vary greatly in composition or content, which is the fundamental reason for the difference in the selection behavior of phytophagous pests toward different plants (Kong and Lou, 2010). Similarly, phytophagous insects do not sense all volatiles. For example, *Smicronyx fulvus* only has a preference for a specific-proportion of five monoterpenes in the odor released by *Helianthus annuus* (Roseland et al., 1992). *Plutella xylostella* is strongly influenced by the mustard oil (Isothiocyanates) emitted by plants of this family when making selections for Brassicaceae plants (Verkerk and Wright, 1994). Farnesene released by *Zea mays* has a strong attractive effect on *Ostrinia nubilalis* (Binder et al., 1995). The odor of *Datura wrightii* flowers consists of about 60 compounds, but *Manduca sexta* only responds behaviorally to nine of them (Riffell et al., 2009). During pollination, *Ceratosolen solmsi* can sense three sesquiterpenes and benzyl benzoate in *Ficus hispida* flowers, whereas *C. gravelyi* can sense 4-methylanisole in *F. semicordata* flowers (Zhang et al., 2020b). The decanal released by *F. pumila* during the pollination stage was significantly attractive to *Wiebesia pumilae* (Wang et al., 2021). In this study, we found that the amount and composition of the volatiles in the three tea species were not significantly correlated, but the relative contents of each component were different. The relative contents of hexanol, linalool, and geraniol, although not high, were significantly and positively correlated with the behavioral preference of *E. onukii*. This correlation was confirmed in single and mixed tests of these three components. These three volatile components could be the specific components that determine the host selection behavior of *E. onukii*, and the results of this study can provide key components for the subsequent development of specific lures for this insect.

Plant volatiles are important components for insects to find hosts and have effects on insects such as luring, repelling, and transmitting information (Schoonhoven et al., 2005). There are about 100 kinds of volatiles in fresh tea leaves, including terpenoids, alcohols, hydrocarbons, aldehydes, ketones, esters, benzenes, phenols, acids, lactones and nitrogenous compounds, which are mainly formed by the pathways such as terpenoid biosynthesis, benzene ring compound synthesis, carotene oxidative degradation, and fatty acid derivative synthesis (Lian et al., 2014). Hexanol is a fatty alcohol compound, which has been reported to have a luring effect on *Melanoplus sanguinipes* (Kang and Hopkins, 2004). In the present study, this compound was also found to have a significant luring effect on *E. onukii* at the tested concentration range, reaching a maximum at 6 µg/100 µL. Linalool and geraniol are monoterpene compounds, of which linalool has been reported to have a significant effect on *E. vitis* (Zhao et al., 2002; Wang et al., 2016), *M. sexta* (He et al., 2019), and *Thrips hawaiiensis* (Li et al., 2022a), whereas geraniol has also been reported to have eliciting effects on *E. vitis* (Chen et al., 2003), *Chrysopa septempunctata* (Han and Zhou, 2004), and *Frankliniella occidentalis* (Li et al., 2022b), both of which were also found to have significant elicitation effects on *E. onukii* in the tested concentration range, reaching a maximum at 12 µg/100 µL and 9 µg/100 µL, respectively. These results not only validated the selection of the three tea volatiles by *E. onukii* but also provided a basis for the concentration configuration of the key components in the subsequent development of this insect-specific lure, as well as a reference for the study of the effect of these components on the behavioral preference of other insects.

The type and content of volatiles produced by plants are genetically controlled (Schoonhoven et al., 2005; Kong and Lou, 2010). Fatty alcohol compounds are synthesized mainly through the lipoxygenase pathway catalyzed by related genes controlling lipoxygenase (LOX), hydroperoxide lyase (HPL), and alcohol dehydrogenase (ADH) (Contreras and Beaudry, 2013; Zhou et al., 2019; Niu et al., 2022; Wang et al., 2022). Monoterpenes are mainly synthesized through the monoterpenoid biosynthesis pathway catalyzed by terpene synthase (TPS) controlled by related genes (Schilmiller et al., 2009; Yu and Utsumi, 2009; Zhou et al., 2019). Among the three volatiles involved in this study, hexanol was reported to be highly significantly correlated with the expression levels of the *LOX1* and *ADH* genes in *C. sinensis* (Zhou et al., 2019) . The hexanol content in *Malus pumila* × *M. asiatica* (Wang et al., 2022) was significantly and positively correlated with the expression of *LOX2a*, and in *Vitis vinifera* it was correlated with the expression of *LOXA*, *HPL1*, and *ADH3* (Li et al., 2022b). The present study also found a significant positive correlation between the relative content of this volatile substance and the expression of *TEA009423 (LOX2.1)*, *TEA009596 (LOX1.5)*, *TEA008699 (HPL)*, *TEA018669 (CYPADH)*, and *TEA015686 (ADHIII)*. Linalool has been previously reported to be significantly correlated with the expression of linalool in the strawberry plants *Fragaria* spp. (Aharoni et al., 2004), *Antirrhinum majus* (Nagegowda et al., 2008), *Gossypium hirsutum* (Huang et al., 2018), *Nicotiana attenuata* (He et al., 2019) and *C*. *sinensis* (Mei et al., 2017) are regulated by genes *FaNES1*, *AmNES/LIS-2*, *GhTPS12*, *NaLIS* and *CsLISs*, respectively. Geraniol has been previously reported in *Ocimum basilicum* (Iijima et al., 2004), *Madagascar periwinkle* (Simkin et al., 2012), *Rosa hybrida* (Magnard et al., 2015), and *Camptotheca acuminata* (Chen et al., 2016), which are regulated by the genes *ObGES, CrGES, RhNUDX1*, and *CaGES*, respectively. The present study found that linalool was significantly and positively correlated with the expression of the genes *TEA001435 (CAD)* and *Camellia_sinensis_newGene_22126 (TPS)*. Although the sequences of these two genes are only 42.75% similar to the identified linalool synthase genes *CsLIS1* and *CsLIS2* in *C*. *sinensis* by Mei et al. (2017), they all belong to the terpene synthase gene and are probably involved in linalool synthesis in *C*. *sinensis*. The relative content of geraniol was significantly correlated with the expression of the genes *TEA001435 (CAD)*, *TEA002658 (CYP76B6)*, *TEA025455 (CYP76T24)*, and *Camellia_sinensis_newGene_22126 (TPS)*. It can be seen that the nine genes identified in this study may be key genes for the synthesis of three specific volatiles in tea, and may provide candidate target genes for the subsequent breeding of tea varieties resistant to *E. onukii*.

Plant volatiles play a crucial role in the host selection of phytophagous insects (Kong and Lou, 2010). Volatiles with luring and repellent effects on phytophagous insects can not only be used to develop specific lures or repellents but also the types and levels of volatiles in plants can be regulated by genetic breeding and genetic engineering technology. The components that lure phytophagous insects can be reduced and the components that repel phytophagous insects can be increased. In addition, the components that lure the natural enemies of phytophagous insects can be amplified. This would maximize the role of plant insect resistance and reduce the damage by phytophagous insects. In this way, we can not only reduce the damage of phytophagous insects by giving full play to plant insect resistance but also protect the ecological environment and achieve the unity of economic, social, and ecological benefits, which is in line with the goal of green and sustainable pest control (Xiang et al., 2015). In this study, Three volatile compounds in tea that have attractive effects on its main pest *E. onukii* were found, and revealed nine genes potentially related to the synthesis of these volatile compounds. But we still need to do lots of work to verify the function of related genes that regulate the VOCs in three tea species. For example, Bate and Riley (1998) confirmed that the gene *ADH1* mutant *Arabidopsis thaliana* release lower amounts of hexanol compared to wild type plant by transient silencing. Mei et al. (2017) confirmed that genes *CsLIS1* and *CsLIS2* produced linalool from geraniol diphosphate in *C*. *sinensis* by enzyme-catalyzed reaction. In the future, we will further explore the optimal ratio of these three volatiles, and verify the functions of genes related to the synthesis of these specific volatiles by enzyme-catalyzed reaction, transgenic plants and transient expression (or silencing) (Mei et al., 2017). This will contribute to the development of *E. onukii* specific attractants and the cultivation of tea insect resistant varieties, promote the development of the "push pull" management strategy for this pest, and provide important basis for the green prevention and control of tea garden pests and the protection of the ecological environment (Mei et al., 2017).

## Data availability statement

The datasets presented in this study can be found in online repositories. The names of the repository/repositories and accession number(s) can be found below: GenBank, OR179901-OR179909.

## Author contributions

J-FH and X-GL designed the study and approved the final draft. FC, PH, and JW conducted the experiments, analyzed the data, and wrote the first draft of the manuscript. WW and Y-WL revised the manuscript and did the supervision. All authors contributed to the article and approved the submitted version.
