# Supplementary figures and images for: Specific volatiles of tea plants determine the host preference behavior of Empoasca onukii
Source: Front Plant Sci. 2023 Aug 31;14:1239237. doi: 10.3389/fpls.2023.1239237 (PMC10501839; doi:10.3389/fpls.2023.1239237)

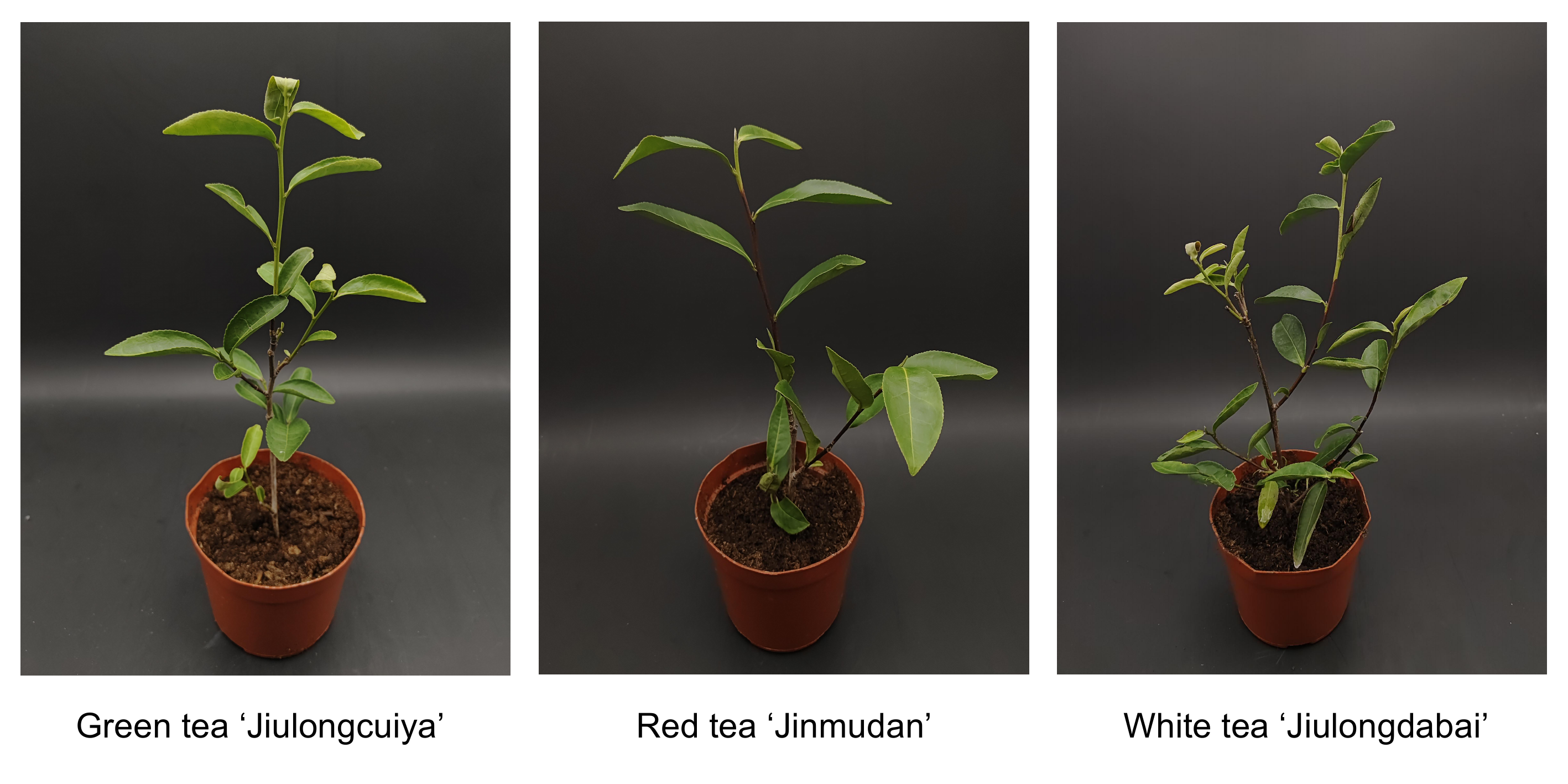

Supplement: Supplementary file 1 [file Image_1.tif]

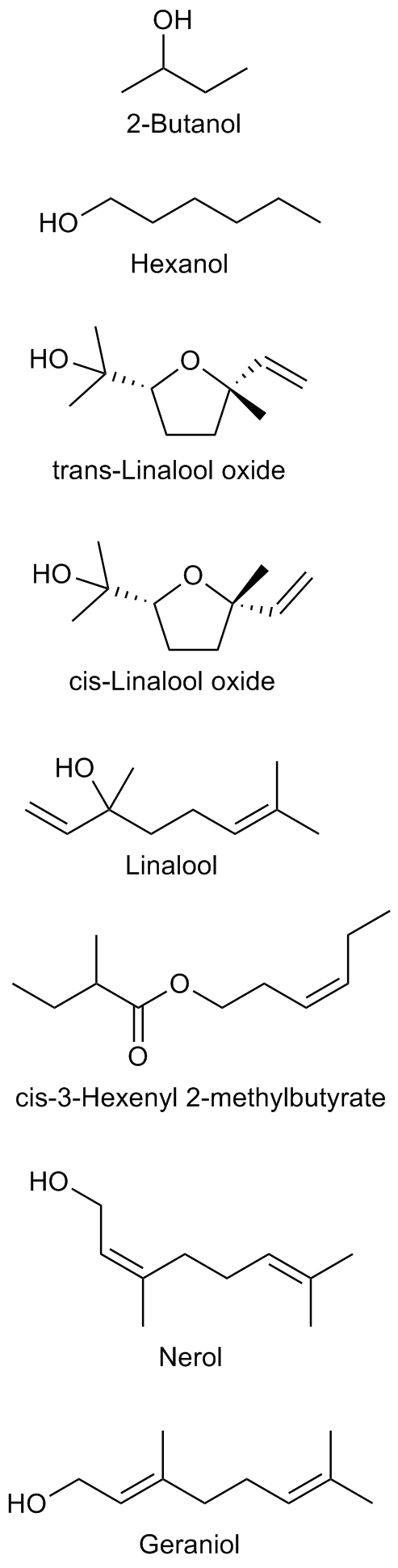

Supplement: Supplementary file 2 [file Image_2.tif]
