# Supplementary material for: Specific volatiles of tea plants determine the host preference behavior of Empoasca onukii
Source: Front Plant Sci. 2023 Aug 31;14:1239237. doi: 10.3389/fpls.2023.1239237 (PMC10501839; doi:10.3389/fpls.2023.1239237)
Supplement: Supplementary file 3 [file Table_1.docx]

Table S1 Quality score and base calling accuracy of the sequencing datas of three potted tea plants

| Phred Quality Score | Probability of incorrect base call | Base call accuracy |
| --- | --- | --- |
| 10 | 1/10 | 90% |
| 20 | 1/100 | 99% |
| 30 | 1/1000 | 99.9% |
| 40 | 1/10000 | 99.99% |

Quality Score or Q-score represents the probability of an incorrect base. This Phred quality score is defined as following equation: Q=−10×log_10_ *P*. In the equation, *P* stands for the base calling error probabilities.

| Samples | Clean reads | Clean bases | GC Content | %≥Q30 |
| --- | --- | --- | --- | --- |
| Green tea treatment 1 | 20,863,921 | 6,235,633,660 | 44.22% | 93.86% |
| Green tea treatment 2 | 20,270,478 | 6,055,392,938 | 44.27% | 94.02% |
| Green tea treatment 3 | 20,310,760 | 6,065,654,510 | 44.40% | 94.22% |
| Red tea treatment 1 | 20,211,674 | 6,039,538,516 | 43.73% | 94.28% |
| Red tea treatment 2 | 19,431,675 | 5,806,005,094 | 43.95% | 94.36% |
| Red tea treatment 3 | 20,888,534 | 6,243,732,954 | 44.30% | 94.41% |
| White tea treatment 1 | 20,688,403 | 6,180,837,420 | 43.83% | 94.10% |
| White tea treatment 2 | 20,594,180 | 6,153,176,038 | 43.66% | 93.70% |
| White tea treatment 3 | 20,583,442 | 6,143,835,546 | 44.16% | 94.38% |

Table S2 Sequencing data Statistics of three potted tea plants

Samples: Sample name. Clean reads: Counts of clean PE reads. Clean bases: total base number of Clean Data. GC content:

Percentage of G, C in clean data. %≥Q30: Percentage of bases with Q-score no less than Q30.

Table S3 Statistics on data mapping of three potted tea plants

| Samples | Total Reads | Mapped Reads | Uniq Mapped Reads |
| --- | --- | --- | --- |
| Green tea treatment 1 | 41,727,842 | 37,873,040 (90.76%) | 35,909,350 (86.06%) |
| Green tea treatment 2 | 40,540,956 | 36,871,416 (90.95%) | 34,973,910 (86.27%) |
| Green tea treatment 3 | 40,621,520 | 36,958,615 (90.98%) | 34,976,503 (86.10%) |
| Red tea treatment 1 | 40,423,348 | 35,798,270 (88.56%) | 33,970,414 (84.04%) |
| Red tea treatment 2 | 38,863,350 | 34,334,023 (88.35%) | 32,547,793 (83.75%) |
| Red tea treatment 3 | 41,777,068 | 37,510,021 (89.79%) | 35,601,976 (85.22%) |
| White tea treatment 1 | 41,376,806 | 37,635,990 (90.96%) | 35,685,890 (86.25%) |

Total Reads: Counts of Clean Reads, counted as single end. Mapped Reads: Counts of mapped reads and the proportion of that in clean data. Uniq Mapped Reads: Counts of reads mapped to a unique position on reference genome and proportion of that in clean data.
